# Seroprevalence and molecular detection of SARS-CoV-2 among apparently healthy healthcare workers and patients in a Nigerian tertiary hospital

**DOI:** 10.1186/s12879-025-12021-y

**Published:** 2025-11-14

**Authors:** Rabi Ahmed Mahdi, Ahmad Hayatu, Umar Mohammed Hassan, Muhammad Sani Aliyu, Muhammad Hassan Isa Doko, Usman Yahaya

**Affiliations:** 1 Ahmadu Bello University Teaching Hospital, Zaria, Kaduna State Nigeria; 2 Federal Teaching Hospital Gombe, Gombe, Gombe State Nigeria; 3 College of Medicine and Vertinary Medicine, The University of Edinburgh, EH16 4SB Edinburgh, Scotland, UK; 4 Ahmadu Bello University, Zaria, Kaduna State Nigeria

**Keywords:** SARS-CoV-2, COVID-19, Seroprevalence, qRT-PCR, Healthcare workers, Nigeria, Infection prevention and control

## Abstract

**Background:**

SARS-CoV-2 continues to drive community and healthcare-associated transmission globally, with limited data from sub-Saharan Africa on asymptomatic carriage and infection dynamics. We conducted a dual serological and molecular study to determine the prevalence of past and current SARS-CoV-2 infections among apparently healthy healthcare workers (HCWs) and patients in a Nigerian tertiary hospital.

**Methods:**

We performed a cross-sectional study at the Federal Teaching Hospital, Gombe, Nigeria, enrolling 250 participants (35 HCWs and 215 patients). Venous blood samples were tested for anti-SARS-CoV-2 IgM/IgG antibodies using validated rapid diagnostic tests, while nasopharyngeal/oropharyngeal swabs were analysed by real-time reverse transcription polymerase chain reaction (qRT-PCR) targeting SARS-CoV-2 genes. Socio-demographic and behavioural data were collected via structured questionnaires. Data were analysed using descriptive statistics and chi-square tests, with significance set at *p* < 0.05.

**Results:**

Overall, 42.0% (*n* = 105) of participants were seropositive for SARS-CoV-2 antibodies, predominantly IgG (41.6%), with minimal IgM detection (0.4%). Antibody prevalence was significantly higher among patients (37.6%) than HCWs (4.0%) (*p* < 0.001). SARS-CoV-2 RNA was detected in 42.8% (*n* = 107) of participants, all of whom were patients. No HCWs tested positive for active infection. All qRT-PCR–positive cases were also IgG positive, suggesting late-phase or resolving infections. Risk factor analysis revealed no statistically significant associations between infection and comorbidities such as hypertension, diabetes, or asthma (*p* > 0.05).

**Conclusions:**

This study demonstrates substantial community transmission of SARS-CoV-2 among apparently healthy patients in a Nigerian tertiary hospital, contrasted with the absence of active infection among HCWs, which may reflect effective IPC practices, although other unmeasured factors could also contribute. The combined use of serology and molecular testing provided complementary insights into both past exposure and ongoing infections. Our findings highlight the importance of dual diagnostic strategies for surveillance in high-risk healthcare settings and reinforce the need for sustained IPC measures, alongside strengthened community-level interventions, to mitigate COVID-19 transmission in Nigeria.

**Clinical trial:**

Not applicable.

**Supplementary Information:**

The online version contains supplementary material available at 10.1186/s12879-025-12021-y.

## Introduction

Severe acute respiratory syndrome coronavirus 2 (SARS-CoV-2), the causative agent of coronavirus disease 2019 (COVID-19), emerged in Wuhan, China, in December 2019 and rapidly spread worldwide. Within weeks, the virus had crossed national borders, prompting the World Health Organization (WHO) to declare COVID-19 a Public Health Emergency of International Concern on 30 January 2020, and subsequently a pandemic on 11 March 2020 [1, 2]. Since its emergence, SARS-CoV-2 has caused profound health, social, and economic impacts globally, with more than 1.2 billion confirmed cases and over 7.1 million reported deaths worldwide as of the time of writing [3]. The virus continues to circulate, with periodic surges driven by emerging variants that demonstrate higher transmissibility or immune evasion [4].

### Virology and transmission dynamics

SARS-CoV-2 is an enveloped, positive-sense, single-stranded RNA virus of the genus *Betacoronavirus* and family *Coronaviridae*. Morphologically, it is characterised by club-shaped spike (S) glycoproteins that protrude from the viral envelope, giving the virus its crown-like appearance under electron microscopy [5]. These spike proteins bind to the angiotensin-converting enzyme 2 (ACE2) receptor on host cells, enabling viral entry and replication within the host cell [6].

Transmission of SARS-CoV-2 occurs mainly via direct contact and respiratory droplets expelled during coughing, sneezing, talking, or breathing, with airborne (aerosol) transmission possible—especially during aerosol-generating medical procedures or in poorly ventilated indoor settings [7]. Indirect transmission via contaminated surfaces (fomites) has been documented, though its relative importance appears lower compared to airborne and droplet spread [8]. Notably, asymptomatic and presymptomatic individuals significantly contribute to SARS-CoV-2 spread. Meta-analyses estimate that about 20% of infected persons remain asymptomatic throughout the course of infection (95% CI: 17–25%), and 31% identified through population screening also remained symptom-free (95% CI: 26–37%) [9, 10]. Epidemiological models estimate that presymptomatic transmission constitutes over 40% of total SARS-CoV-2 spread, underscoring its major role compared to asymptomatic carriers, who account for less than 15% [9]. One modeling study estimated that asymptomatic individuals exhibited 66.7% lower transmissibility compared to symptomatic cases, yet comprised 28.2% of all infections and potentially played an increasing role in ongoing community transmission [11]. This silent transmission underscores the need for surveillance strategies that detect infections beyond symptomatic cases.

### Role of seroprevalence and molecular detection

Seroprevalence studies, which detect antibodies such as immunoglobulin M (IgM) and immunoglobulin G (IgG) against SARS-CoV-2, are essential for estimating the proportion of a population previously exposed to the virus [12]. IgM antibodies against SARS-CoV-2 typically rise early during infection, often becoming detectable within the first week of symptom onset, whereas IgG antibodies generally appear around 10–14 days post-onset and can remain detectable for several months [13, 14]. Although IgG presence does not guarantee sterilising immunity, it serves as a reliable epidemiological marker of past exposure to the virus [13].

In contrast, real-time reverse transcription polymerase chain reaction (qRT-PCR) remains the gold standard for diagnosing active SARS-CoV-2 infection by detecting viral RNA in respiratory specimens. This method is sensitive, specific, and widely accepted globally [15]. By amplifying viral genetic material, qRT-PCR enables accurate identification of ongoing infection, which is critical for guiding isolation, contact tracing, and outbreak containment strategies. When combined with serological testing, which provides insight into past exposure, researchers and clinicians gain a comprehensive epidemiological perspective on both current infection and historical transmission dynamics [16].

### SARS-CoV-2 in healthcare settings

Healthcare workers (HCWs) are a high-risk occupational group due to frequent exposure to COVID-19 patients—including those with undiagnosed infection—as well as their involvement in aerosol-generating procedures (AGPs). Systematic reviews have demonstrated that HCWs exposed to AGPs face 1.7 to 2.5 times higher odds of infection [17, 18]. The early pandemic period saw high infection rates among HCWs, primarily driven by shortages of personal protective equipment (PPE), inadequate infection prevention and control (IPC) training, and limited diagnostic testing capacity [19]. Conversely, rigorous IPC protocols—including proper donning and doffing of PPE, hand hygiene, and environmental disinfection—have been shown to substantially reduce the risk of transmission in healthcare [15, 17].

Patients who are asymptomatic or pre-symptomatic can contribute significantly to nosocomial transmission if they go undetected and un-isolated [20]. For instance, in a skilled nursing facility, more than half of residents who Although these individuals were asymptomatic when tested, viable virus was later isolated from those who went on to develop symptoms, demonstrating that transmission can occur during the presymptomatic phase [21]. This risk is particularly pronounced in high-turnover settings such as emergency departments and outpatient clinics, where symptom-based screening may fail to identify silent carriers. Proactively detecting and isolating individuals who are presymptomatic (infected but not yet symptomatic) and those who remain asymptomatic is essential for protecting healthcare workers and vulnerable patients, since both can shed virus and facilitate transmission.

### Nigerian epidemiological context

Nigeria confirmed its first COVID-19 case on 27th February, 2020, involving an Italian citizen who returned from Milan, Italy, underscoring early gaps in airport surveillance and preparedness. Since that time, the country has experienced multiple waves of varying intensity across its 36 states and the Federal Capital Territory [22–24]. While national testing capacity has greatly expanded—from just five laboratories in early 2020 to over 70 by February 2021—under-detection and under-reporting remain significant challenges, particularly in rural areas where access to diagnostic services and surveillance tools is limited [24, 25].

Several studies in Nigeria have assessed SARS-CoV-2 seroprevalence, revealing considerable regional and temporal variation [26, 27]. Estimated seroprevalence across four states in October 2020: 9.3% in Gombe (95% CI: 7.0–11.5), 25.2% in Enugu (95% CI: 21.8–28.6), 23.3% in Lagos (95% CI: 20.5–26.4), and 18.0% in Nasarawa (95% CI: 14.4–21.6) [26, 28]. A serosurvey conducted in Kaduna State during October–November 2021 and reported overall seroprevalence at 43.7%, with higher rates in rural sites (53.5%) compared to urban (42.5%) [27]. Together, these data underscore substantial heterogeneity in SARS-CoV-2 exposure within Nigeria, influenced by geographic location, timing, and population characteristics.

Although these studies provide valuable snapshots of SARS-CoV-2 exposure, few have incorporated both serological and molecular testing to assess past and active infections concurrently. This dual approach is particularly important in identifying ongoing transmission among asymptomatic individuals and evaluating IPC measures in healthcare environments.

### Rationale for the present study

Federal Teaching Hospital, Gombe, is a major tertiary referral centre for the North-East geopolitical zone of Nigeria, serving a diverse population and employing a wide range of HCWs across various clinical departments. Given the high volume of patient interactions, including referrals from rural and peri-urban areas, the hospital represents an important site for epidemiological surveillance.

Assessing SARS-CoV-2 prevalence among apparently healthy HCWs and patients in this setting addresses several critical knowledge gaps:

1. Infection risk assessment for HCWs – Understanding whether IPC measures are effectively protecting staff from occupational exposure.
2. Detection of asymptomatic carriers – Identifying patients with active infection who may unknowingly contribute to nosocomial transmission.
3. Public health planning – Informing hospital and state-level policy on screening, isolation, and resource allocation.

By integrating serological and molecular diagnostic methods, this study provides a more complete understanding of the burden of both past and current SARS-CoV-2 infections in a high-risk hospital environment.

### Aim and objectives

The overarching aim of this study was to determine the seroprevalence and molecular detection of SARS-CoV-2 among apparently healthy HCWs and patients in a Nigerian tertiary hospital. The specific objectives were to:

1. Determine the prevalence of anti-SARS-CoV-2 IgM and IgG antibodies in the study population.
2. Detect active SARS-CoV-2 infection using qRT-PCR.
3. Assess associations between infection status and socio-demographic or behavioural risk factors.

## Methods

### Study design and setting

This study employed a cross-sectional design to determine the seroprevalence and molecular detection of SARS-CoV-2 among apparently healthy healthcare workers (HCWs) and patients at the Federal Teaching Hospital, Gombe (FTHG), Nigeria. FTHG is a tertiary referral centre serving the North-East geopolitical zone, with a capacity of over 500 beds and a diverse range of clinical departments. The study was conducted during a period of ongoing community transmission of SARS-CoV-2 in the region.

### Study population

The target population comprised two groups:

1. Healthcare workers — including doctors, nurses, laboratory scientists, and ancillary staff with direct or indirect patient contact.
2. Patients — outpatients and those attending specialist clinics, excluding those with overt symptoms suggestive of COVID-19 at the time of recruitment.

Participants were considered *apparently healthy* if they had no fever, cough, shortness of breath, or other symptoms suggestive of acute COVID-19 in the preceding two weeks.

### Sample size determination

Sample size was calculated using the Cochran formula for cross-sectional studies [29]. Based on an anticipated SARS-CoV-2 antibody prevalence of 20%, a 95% confidence level, and a 5% margin of error [28]. The final sample included 250 participants, proportionally distributed between HCWs and patients according to their relative numbers in the hospital.

1 $${{\text{n}}_{\text{0}}}\,{\text{ = }}\,\frac{{{{\text{Z}}^{\text{2}}}{\text\,{pq}}}}{{{{\text{e}}^{\text{2}}}}}$$

Where: ${{\text{n}}_{\text{0}}}$= Minimum number of samples required (sample size)

Z = Standard normal deviate at 95% confidence interval = 1.96

p = estimated proportion of respondents with SARS-CoV-2 antibodies (0.20)

e = Level of precision (0.05%)

q = 1-p

### Sampling technique

A stratified random sampling technique was applied to ensure proportional representation of key subgroups. Healthcare workers (HCWs) were stratified according to professional category (e.g., physicians, nurses, laboratory staff, and support staff), while patients were stratified based on clinic type (general outpatient, specialty clinics, and emergency services). Within each stratum, participants were selected using simple random sampling until the predetermined sample size for that stratum was achieved.

### Data collection tools and procedure

Data were collected using a structured, interviewer-administered questionnaire adapted from the World Health Organization’s COVID-19 sero-epidemiological investigation protocols [30]. The instrument was pre-tested in a pilot study at the same facility to assess clarity, cultural appropriateness, and ease of administration, and subsequently refined based on feedback from participants and interviewers. The questionnaire captured information on socio-demographic characteristics, occupation, travel history, contact with confirmed COVID-19 cases, mask-use practices, and other relevant behavioural and clinical risk factors. Data collection took place between July and August 2022.

Following informed consent, each participant provided:

1. Venous blood sample (5 mL**)** for antibody testing.
2. Nasopharyngeal and oropharyngeal swabs for qRT-PCR.

Specimen collection was performed by trained laboratory scientists using standard biosafety procedures.

### Laboratory analysis

#### Serological testing

Serum was separated from clotted blood and tested for anti-SARS-CoV-2 IgM and IgG antibodies using Clarity COVID-19 IgG/IgM Rapid Test Cassette (Whole blood/ serum/ plasma), by Clarity Diagnostic, previously validated and results were interpreted according to the manufacturer’s instructions [31]:

- IgM positive only — suggestive of recent infection.
- IgG positive only — suggestive of past infection.
- Both IgM and IgG positive — suggestive of ongoing or resolving infection.
- Negative — no detectable antibodies.

#### Molecular testing

RNA was extracted from swab samples according to the manufacturer’s instructions. Detection of SARS-CoV-2 was carried out via real-time reverse transcription polymerase chain reaction (qRT-PCR) using primers and probes targeting the genes, following the Berlin protocol (Corman et al., 2020). Amplification was performed on Applied Biosystems 7500 Real-Time PCR System™ and results were classified as positive, negative, or indeterminate based on cycle threshold (Ct) values and the shape of the amplification curves.

### Data management and analysis

Data from questionnaires and laboratory results were entered into Microsoft Excel and analysed using Statistical Package for the Social Sciences (SPSS) version 26. Descriptive statistics (frequencies, proportions, means, and standard deviations) were computed for socio-demographic variables and prevalence rates. Chi-square was used to assess associations between categorical variables. Statistical significance was set at *p* < 0.05.

### Ethical considerations

#### Ethical approval

was obtained from the Research and Ethics Committee of the Federal Teaching Hospital, Gombe (approval number NHREC/25/10/2013). Participation was voluntary, with informed consent obtained from all participants. Data confidentiality was maintained by using anonymised identifiers, and all procedures adhered to the Declaration of Helsinki.

## Results

### Sociodemographic characteristics of participants

Table 1 presents the socio-demographic characteristics of the study population A total of 250 participants were enrolled, comprising 35 [14%] HCWs and 215 [86%] patients. Participants’ ages ranged from 6 to 75 years. The largest age group was 26–35 years (*n* = 102), while the smallest was 6–15 years (*n* = 2). The 26–35-year age group also recorded the highest proportion of COVID-19 infection, with 46 individuals (48.6%) testing positive.

Regarding marital status, married participants were the most represented (*n* = 165) and had the highest number of COVID-19 infections, with 78 individuals (73.3%) testing positive. By occupation, individuals engaged in business recorded the highest infection prevalence (48.2%). Similarly, participants with a tertiary level of education showed the highest proportion of COVID-19 infection (47.2%).

**Table 1:** Sociodemographic characteristics of participants

Variable	No examined	Number Positive (%)
**Age (years)**		
6–15	2	0 (0)
16–25	83	30 (27.6)
26–35	102	52 (48.6)
36–45	47	16 (15.2)
46–55	7	4 (3.8)
56–65	4	1 (1.0)
66–75	5	4 (3.8)
Total	250	107 (36.8)
**Marital status**		
Single	81	28 (25.7)
Married	165	78 (73.3)
Divorced	3	1 (1.0)
Widowed	1	0 (0)
Total	250	107 (36.8)
**Educational level**		
Informal	28	13 (12.4)
Primary	10	5 (4.8)
Secondary	70	38 (35.2)
Tertiary	142	51 (47.6)
Total	250	107 (36.8)
**Occupation**		
Business	85	52 (48.6)
Civil servants	78	17 (16.2)
Pupil	2	0 (0)
Students	41	23 (21.0)
Unemployed	44	15 (14.3)
Total	250	107 (36.6)

### Seroprevalence of SARS-CoV-2 antibodies

Table 2 summarises antibody prevalence by participant group and antibody type. Overall, 105 (42.0%) of participants tested positive for SARS-CoV-2 antibodies. IgG antibodies were detected in 104 (41.6%), while IgM antibodies were detected in only 1 (0.4%). None of the participants had both IgM and IgG positive results simultaneously. Among HCWs, antibody prevalence was 11 (4.4%), while patient antibody prevalence was significantly higher at 94 (37.6%).

**Table 2:** SARS-CoV-2 antibody prevalence by participant group and antibody type

Antibody status	HCW (*n* = 29)	Patients (*n* = 221)	Total (*n* = 250)
IgG	10 (34.5%)	94 (42.5%)	104 (41.6%)
IgM	1 (3.4%)	0 (0.0%)	1 (0.4%)
Negative	18 (62.1%)	127 (57.5%)	145 (58.0%)

### Molecular detection of SARS-CoV-2 (qRT-PCR)

SARS-CoV-2 RNA was detected in 107 (42.8%) of participants (all of whom were patients), all of whom were patients. No HCWs tested positive for active infection (Table 3).

**Table 3:** Detection of SARS-CoV-2 RNA by qRT-PCR among study participants

qRT-PCR result	HCWs (*n* = 29)	Patients (*n* = 221)	Total (*n* = 250)
Positive	0 (0.0%)	107 (48.4%)	107 (42.8%)
Negative	29 (100.0%)	114 (51.6%)	143 (57.2%)

### Risk factors for seropositivity and PCR positivity

Table 4 provides details of predictors of seropositivity and PCR positivity. Out of 250 participants, 107 (42.8%) tested positive for COVID-19. Reported prevalence of investigated risk factors was low: tobacco smoking (*n* = 1, 0.4%; *p* = 0.179), alcohol consumption (*n* = 2, 0.8%; *p* = 0.698), asthma (*n* = 1, 0.4%; *p* = 0.642), diabetes mellitus (*n* = 2, 0.8%; *p* = 0.227), cancer (*n* = 3, 1.2%; *p* = 1.000), HIV infection (*n* = 0; *p* = 1.000), and hypertension (*n* = 20, 8.0%; *p* = 1.108). None of these factors were significantly associated with COVID-19 infection (*p* > 0.05 for all). There were no statistically significant associations between infection status and common comorbidities or lifestyle factors (all *p* > 0.05).

**Table 4:** Predictors of seropositivity and PCR positivity

Symptoms	Number examined	Number positive (%)	ꭓ^2^	*P* = value
**Smoking tobacco**				
Yes	1	0(0)	1.803	0.1794
No	249	107(100)		
**Consumption of alcohol**				
Yes	2	1(1.0)	0.1510	0.6975
No	248	106(99.0)		
**Asthma**				
Yes	1	0(0)	0.215	0.642
No	249	107(100)		
**Diabetes Mellitus**				
Yes	2	1(1.0)	1.460	0.227
No	248	106(99.0)		
**Cancer**	250		0.000	1.000
Yes	3	0(0)		
No	247	107 (100)		
**Hypertension**			2.579	1.108
Yes	20	3(2.9)		
No	230	102(97.1)		

## Discussion

### Comparison with other Nigerian studies

The seroprevalence observed in our study exceeds some earlier Nigerian reports but aligns with others. Reported rates of 9.3% in Gombe, 25.3% in Enugu, 23.3% in Lagos, and 18.1% in Nasarawa, with the lower Gombe figure likely reflecting the earlier timing of their study [26]. In Kaduna State, the prevalence was found to be 42.5% (IgG 36.75%, IgM 10.25%), similar to our total antibody prevalence but with higher IgM, suggesting more recent infections [27]. Likewise, a prevalence of 45.1% was reported in Ibadan, highlighting substantial SARS-CoV-2 exposure in Southwestern Nigeria [32].

Conversely, a large-scale study in Anambra State involving 3,142 participants across 21 local government areas found a markedly higher prevalence of 66.7% [33]. This difference may be explained by variations in sample size, population demographics, timing relative to epidemic waves, and local outbreak severity.

Our IgM prevalence (0.4%) is notably lower than in other Nigerian studies [27]. The low IgM detection in our cohort suggests minimal ongoing or very recent infection at the time of sampling, consistent with the lack of active infection among HCWs by qRT-PCR.

### Comparison with African and global data

Across Africa, reported seroprevalence varies widely depending on study timing and location. Seropositivity in Zambia of 10.6% was reported among HCWs, and in Kenya was found to be 4.3% in blood donors early in the pandemic [34, 35]. Later Kenyan studies, however, observed rates exceeding 40% as the pandemic progressed. In South Africa, documented seroprevalence over 50% in certain provinces following the Beta variant wave [36]. These patterns illustrate how timing relative to epidemic peaks profoundly influences measured prevalence.

Globally, our seroprevalence falls within the broad range reported in high-burden settings during later pandemic phases, though it remains lower than those observed in India (67.6% after the Delta wave) and Brazil (76% in Manaus following intense transmission) [37, 38]. The lower rates in our study may reflect Nigeria’s generally lower reported case incidence, possibly due to under-ascertainment, younger population demographics, or other contextual factors [39].

### Implications for healthcare workers

A key finding of this study was the absence of active SARS-CoV-2 infection among healthcare workers (HCWs) despite substantial patient positivity. This suggests that infection prevention and control (IPC) measures—including proper use of personal protective equipment (PPE), hand hygiene, and environmental decontamination—were effectively implemented within the hospital. Previous Nigerian studies reported significant infection rates among HCWs, particularly early in the pandemic when PPE shortages were prevalent [24]. By contrast, our findings align with settings where IPC measures were strictly enforced. Nonetheless, the 4.0% antibody positivity among HCWs indicates prior exposure. Given that asymptomatic infection is well documented in this population [40]. These results underscore the importance of ongoing surveillance. Continuous training, adequate PPE supply, and rapid testing remain critical for maintaining low transmission rates among HCWs, especially as new variants emerge.

### Patient infection dynamics

The detection of active infection exclusively among patients points to ongoing community transmission and underscores the importance of pre-admission screening and triage protocols. Many patients may have been asymptomatic carriers, as our inclusion criteria required participants to be apparently healthy at enrolment. Asymptomatic and pre-symptomatic transmission is estimated to contribute up to 30% of SARS-CoV-2, representing a persistent challenge in healthcare settings [41].

The higher seroprevalence among patients compared to HCWs also suggests greater community exposure, possibly linked to lower adherence to preventive measures outside healthcare facilities. The higher seroprevalence among patients compared to HCWs also suggests greater community exposure, possibly linked to lower adherence to preventive measures outside healthcare facilities. Although formal risk-factor modelling was not performed, this pattern is consistent with global evidence emphasising the protective effect of mask-wearing and the increased vulnerability of older individuals to infection [42, 43].

### Serology versus molecular testing

Our combined serological and qRT-PCR approach provided complementary insights into SARS-CoV-2 infection status. Serology captured cumulative exposure, while molecular testing identified current infections. The detection of active infection exclusively among patients indicates ongoing community transmission and underscores the importance of pre-admission screening and triage protocols. Many patients may have been asymptomatic carriers, as all participants were apparently healthy at enrolment. Asymptomatic and pre-symptomatic transmission is estimated to contribute up to 30% of SARS-CoV-2 spread [41], representing a persistent challenge in healthcare settings. Interestingly, all PCR-positive cases were also IgG positive, suggesting these infections were likely in later stages, with immune responses already mounted. The low IgM detection, despite substantial PCR positivity, may reflect the rapid class-switching from IgM to IgG observed in SARS-CoV-2 infections [40].

### Temporal and epidemiological context

The timing of this study is crucial in interpreting its findings. By the time of data collection, Nigeria had experienced multiple waves of COVID-19, including those driven by more transmissible variants. Population mobility, mass gatherings, and limited vaccine coverage likely contributed to sustained community transmission. The high antibody prevalence among patients reflects this cumulative exposure.

The interpretation of serological markers is increasingly influenced by vaccination status. COVID-19 vaccines elicit humoral responses primarily directed against the spike protein, which may result in detectable IgG antibodies even in the absence of prior infection. Studies indicate that vaccination can cause persistently high antibody titres and may complicate differentiation between infection- and vaccine-induced seropositivity when spike-based assays are used [44]. In our study, although vaccination status was not recorded, the relatively low vaccine coverage in Nigeria during the study period suggests that most antibody responses likely reflected natural infection. Nonetheless, future serosurveys should incorporate assays targeting both spike and nucleocapsid proteins to enable clear distinction between vaccine and infection-derived immunity.

In addition, vaccine-induced antibodies may have contributed to seropositivity, although our questionnaire did not specifically capture vaccination status. Given Nigeria’s relatively low vaccination rates during much of the pandemic [45], natural infection is likely to have been the predominant driver of seropositivity in our cohort.

### Strengths of the study

A major strength of this study is its dual-modality testing strategy, which allowed for the detection of both past and current SARS-CoV-2 infections. Many seroprevalence studies that rely solely on antibody testing may miss active cases. Another strength is the focus on apparently healthy individuals, providing valuable insights into asymptomatic carriage in high-risk environments. The inclusion of both healthcare workers (HCWs) and patients enables a direct comparison between occupational and community-linked exposure risks, highlighting the relative effectiveness of hospital infection prevention and control (IPC) measures. Additionally, the use of standardized specimen collection procedures and validated assays enhances the reliability and reproducibility of our findings.

### Limitations

As a cross-sectional design, it captures a single time point and cannot track changes in infection or antibody status over time; longitudinal follow-up would be necessary to assess antibody persistence and reinfection risk. The use of a rapid antibody test, while practical, may underestimate early infections or yield false positives due to cross-reactivity with other coronaviruses. The absence of viral sequencing data limits our ability to identify the SARS-CoV-2 variant(s) circulating during the study period. Given the emergence of immune-evasive variants such as Omicron, variant-specific prevalence data would provide valuable epidemiological context. In addition, the reliance on self-reported data represent potential sources of bias that may have influenced our findings. Finally, the lack of vaccination information prevents differentiation between vaccine- and infection-induced antibodies, although natural infection was likely the predominant contributor to seropositivity given low vaccine uptake at the time.

The continuous evolution of SARS-CoV-2 has implications for diagnostic performance. Emerging variants, particularly those with spike or nucleocapsid mutations, may alter assay sensitivity or lead to diagnostic escape [46]. Such mutations can reduce binding efficiency of both PCR primers and antibody-based assays. Because our molecular detection was based on primer sets validated early in the pandemic, waning sensitivity against newer variants cannot be excluded. This limitation underscores the importance of periodic validation of diagnostic reagents as the virus evolves.

### Challenges in COVID-19 diagnosis

Diagnosing COVID-19 remains challenging due to the wide clinical spectrum and limitations of existing tests [47]. Though highly specific, qRT-PCR, is affected by sampling technique, viral load variability, and timing of specimen collection relative to infection onset. Similarly, serological tests are constrained by delayed antibody appearance and possible cross-reactivity with endemic human coronaviruses. In low-resource settings like Nigeria, logistical constraints such as reagent shortages, cold-chain issues, and inconsistent access to molecular platforms further limit timely diagnosis. These barriers emphasize the need for affordable, multiplex diagnostic approaches capable of detecting multiple SARS-CoV-2 antigens or genomic regions.

### Public health relevance

Our findings have direct implications for hospital and public health policy. The absence of active infection among healthcare workers (HCWs) underscores the effectiveness of sustained investment in infection prevention and control (IPC) measures. Conversely, the high positivity rates among patients highlight the need for robust screening and isolation protocols to prevent nosocomial transmission. Public health messaging should continue to emphasize consistent mask use, particularly among older adults and individuals with known exposures. Given the substantial seroprevalence observed in our patient cohort, integrating routine SARS-CoV-2 testing into pre-admission workflows—especially in high-burden areas—could help mitigate in-hospital outbreaks and protect vulnerable patients.

### Future research directions

Further research should investigate the durability of SARS-CoV-2 antibodies in Nigerian populations, including the effects of hybrid immunity resulting from natural infection and vaccination. Studies incorporating genomic sequencing would provide valuable insights into variant-specific transmission dynamics and potential immune escape. Additionally, qualitative research examining barriers to infection prevention and control (IPC) compliance among patients could inform the design of targeted behavioural interventions.

## Conclusion

In summary, this study adds to the growing evidence on SARS-CoV-2 epidemiology in Nigerian healthcare settings. The combination of high antibody prevalence among patients, absence of active infection among healthcare workers (HCWs), and low IgM detection suggests that, while community transmission remains substantial, in-hospital infection prevention and control (IPC) measures are largely effective. Sustained vigilance, alongside strengthened community-level preventive strategies, is essential to mitigate ongoing transmission risks.

## Supplementary Information

Below is the link to the electronic supplementary material.

Supplementary Material 1

## Data Availability

The data that support the findings of this study are available on request from the corresponding author. The data are not publicly available due to privacy or ethical restrictions.

## Abbreviations

ACE2: Angiotensin-Converting Enzyme 2
AGMPs: Aerosol-Generating Medical Procedures
AGPs: Aerosol-Generating Procedures
CI: Confidence Interval
COVID-19: Coronavirus Disease 2019
Ct: Cycle Threshold
ELISA: Enzyme-Linked Immunosorbent Assay
FTHG: Federal Teaching Hospital Gombe
HCWs: Healthcare Workers
HIV: Human Immunodeficiency Virus
IgG: Immunoglobulin G
IgM: Immunoglobulin M
IPC: Infection Prevention and Control
NHREC: National Health Research Ethics Committee
PPE: Personal Protective Equipment
qRT-PCR: Quantitative Real-Time Reverse Transcription Polymerase Chain Reaction
RNA: Ribonucleic Acid
SARS-CoV-2: Severe Acute Respiratory Syndrome Coronavirus 2
SPSS: Statistical Package for the Social Sciences
WHO: World Health Organization
