# Supplementary material for: Seroprevalence and molecular detection of SARS-CoV-2 among apparently healthy healthcare workers and patients in a Nigerian tertiary hospital
Source: BMC Infect Dis. 2025 Nov 14;25:1585. doi: 10.1186/s12879-025-12021-y (PMC12619265; doi:10.1186/s12879-025-12021-y)
Supplement: Supplementary file 1 — Supplementary Material 1 [file 12879_2025_12021_MOESM1_ESM.docx]

**QUESTIONAIRE**

**Ethical Approval:**
This study, titled “Seroprevalence and Molecular Detection of SARS-CoV-2 Among Apparently Healthy Healthcare Workers and Patients in a Nigerian Tertiary Hospital”, Ethical approval was obtained from the Research and Ethics Committee of the Federal Teaching Hospital, Gombe (approval number NHREC/25/10/2013).

**Participant Consent:**
Participation in this study is entirely voluntary. The information collected will be kept strictly confidential and used only for research purposes. No names or personal identifiers will appear in any report or publication.

By agreeing to participate and completing this questionnaire, you are providing your informed consent. You may choose not to answer any question you are uncomfortable with, and you may withdraw from the study at any time without any consequences to your care.

If you have questions about your rights as a participant, you may contact the Health Research Ethics Committee of Ahmadu Bello University Teaching Hospital.

**Declaration of Consent:**
☐ I have read (or had read to me) the information above.
☐ I understand that my participation is voluntary.
☐ I consent to participate in this study.

Signature/Thumbprint of Participant: ___________________
Date: ___________________

Signature of Researcher: ___________________
Date: ___________________

SECTION A; SOCIO-DEMOGRAPHICS

1. Age:
2. Gender: male ( ) female ( )
3. Marital status:

Single ( ), Married ( ), Divorced ( ), Widowed ( )

1. Occupation:

Student ( ), Civil Servant ( ), Self-employed ( ), Unemployed ( ), Business ( ).

1. SECTION B; PRESENTING SYMPTOMS AND COVID DIAGNOSIS (tick all that apply)

Fever ( )

Cough ( )

Chest pain ( )

Loss of smell/taste ( )

Others ( )

1. Have you been diagnosed with COVID-19 before?

Yes ( )

No ( )

1. SECTION C: MEDICAL HISTORY (tick all that apply)

Hypertension

Asthma

Diabetes

HIV

Cancer

1. RESULT (To be filled by researchers only)

RDT ( )

PCR ( )
